# Aneurysm

**Published:** 1908-04-11

**Authors:** 


### Points in Surgery.

### ANEURYSM.

### VARIETIES AND SYMPTOMS.

Several varieties of aneurysm are described, according to the effect produced upon the vessel wall by the causes enumerated in the preceding article. Thus, if the cause be a general one, such as an increased blood-pressure, all coats of the artery may yield to the same degree in its entire periphery. The result is known as a fusiform aneurysm. At first all the coats of the vessel are represented in its wall, but later, as the pressure increases, the vessel wall may be so thinned out that the aneurysm is lined in places only by new-formed fibrous tissue. This is more commonly the case in the next variety, the sacculated aneurysm. This starts as a localised dilatation on one aspect only of the circumference of the artery at a spot where some weakness has already existed, most commonly an atheromatous ulcer. In this case the inner coat of the artery is already gone, and as pressure increases the middle and outer coats are squeezed out of existence and the boundaries of the aneurysm come to consist only of fibrous tissue which is the outcome of connective tissue reaction. By some authors the terms "true" and "false" have been used to denote these two varieties respectively; the terms are not good because they do not convey a true idea of the pathological conditions. If an aneurysm is an aneurysm at all, it must necessarily be a true aneurysm, and there is therefore no real distinction implied by the terms. In certain rarer cases a dilatation may make its appearance at the site of an atheromatous patch, and the blood may make its way between the endothelial and muscular coats of the artery. Such a condition is known as a dissecting aneurysm. This is not only a very rare condition, but one which it is impossible to differentiate by any known clinical means. It is therefore only a pathological curiosity.

Injury to a vessel wall may lead to an aneurysm. What happens is that, after a punctured wound, blood leaks out into the neighbouring tissues and becomes encysted by connective tissue reaction. The connection between this extravasation and the parent stem remains patent, and the result is a blood tumour communicating with an artery. As might be expected from the foregoing account, traumatic aneurysms are usually met with in the superficial arteries which are exposed to injury. They rarely attain to large dimensions. The writer remembers seeing a traumatic aneurysm in the radial artery of a carpenter just above the wrist. Some weeks previously he had accidentally wounded himself in the wrist with a bradawl, and when seen he had a typical aneurysm of the radial artery as large as a walnut. It was dissected out comparatively easily after ligature of the parent stem above and below, and he had no further trouble.

How may the presence of an aneurysm be recognised? In a typical case the signs to which it gives rise and the symptoms which it causes are classical. In the first place a tumour is found lying in the course of an artery, and this tumour is pulsating and expansile. Its expansile nature is the most characteristic thing about an aneurysm. It is sometimes hard to tell whether a tumour is expansile or not. The difficulty may often be solved by placing upon its surface two wooden stethoscopes of the old-fashioned pattern. The chest pieces are placed flat upon the tumour, and if it is expansile the ear pieces will be seen to diverge from each other appreciably at each heart beat, because the movements are magnified. On auscultation a bruit which is synchronous with the cardiac systole can be heard over the aneurysm itself and often along the line of the artery beyond the tumour. The pulse in the affected vessel is diminished and delayed when compared with the pulse in the corresponding artery of the opposite side. Pressure on the parent stem above the aneurysm causes the tumour to shrink and become less tense, but it fills again to its original size in two or three beats as soon as the pressure is removed. Conversely the immediate effect of pressure below the tumour is to cause it to become more tense; and finally an aneurysm is generally associated sooner or later with symptoms due to pressure, œdema of the part owing to compression of a vein or pain referred along adjacent nerve trunks. If the aneurysm is situated in a part which is not accessible, the pain may be referred in a most misleading manner. For example, pain in one arm may be the first symptom of an aneurysm in the thorax, and if this fact is not kept constantly in mind much misdirected ingenuity may be spent in making diagnoses to fit the case, while the true state of affairs is overlooked; and, moreover, much valuable time may be lost if, as has been done before now, the case be labelled as one of post-influenzal neuritis and treated accordingly.

Once an aneurysm has formed, its tendency is to increase in size progressively and press surrounding structures out of its way; and it does so because the pressure inside is relatively very great. The principle is somewhat analogous to that of the Bramah press. It is really a question of dynamics: the pressure inside the aneurysm being equivalent to the length of the blood stream from the heart multiplied by the area of the aneurysm in cross section. If one appreciates how great this is, one can understand the comparative ease with which an aneurysm can erode even bone, a structure which in the ordinary way is highly resistant to the effects of pressure. Notwithstanding this, aneurysms occasionally undergo spontaneous cure. The sac fills with blood which clots; under favourable conditions the clot organises and gradually extends into the parent stem and blocks it completely, or the same result may ensue if a small portion of the clot becomes separated and impacted in the artery lower down. In either case the artery from which the aneurysm springs is obliterated, and it is essential that this should happen before an aneurysm can be regarded as cured.

